# Impact of COVID-19 on Cardiovascular Disease Presentation, Emergency Department Triage and Inpatient Cardiology Services in a Low- to Middle-Income Country – Perspective from a Tertiary Care Hospital of Pakistan

**DOI:** 10.5334/gh.1084

**Published:** 2021-12-22

**Authors:** Ghufran Adnan, Pirbhat Shams, Maria A. Khan, Jamshed Ali, Nasir Rahman, Fateh Ali Tipoo, Zainab Samad, Saulat Hasnain Fatimi, Saira Bukhari, Osman Faheem

**Affiliations:** 1 Section of Cardiology, Department of Medicine, The Aga Khan University Hospital, Karachi, PK; 2 Epidemiology and Biostatistics Department of Biostatistics and Epidemiology, The Aga Khan University Hospital, Karachi, PK; 3 Section of Cardiology, Department of Medicine, Aga Khan University, PK; 4 Section of Cardiothoracic Surgery, Department of Surgery, Aga Khan University Hospital, Karachi, PK

**Keywords:** COVID-19, Cardiovascular diseases, Epidemiology, Low-Middle Income Country, Global Health, Cardiovascular Intervention

## Abstract

**Aims:**

To identify the changes in cardiovascular disease presentation, emergency room triage and inpatient diagnostic and therapeutic pathways.

**Methods:**

We conducted a retrospective cohort study at the Aga Khan University Hospital, Karachi. We collected data for patients presenting to the emergency department with cardiovascular symptoms between March–July 2019 (pre-COVID period) and March–July 2020 (COVID period). The comparison was made to quantify the differences in demographics, clinical characteristics, admission, diagnostic and therapeutic procedures, and in-hospital mortality between the two periods.

**Results:**

Of 2976 patients presenting with cardiac complaints to the emergency department (ED), 2041(69%) patients presented during the pre-COVID period, and 935 (31%) patients presented during the COVID period. There was significant reduction in acute coronary syndrome (ACS) (8% [95% CI 4–11], p < 0.001) and heart failure (↓6% [95% CI 3–8], p < 0.001). A striking surge was noted in Type II Myocardial injury (↑18% [95% CI 20–15], p < 0.001) during the pandemic. There was reduction in cardiovascular admissions (coronary care unit p < 0.01, coronary step-down unit p = 0.03), cardiovascular imaging (p < 0.001), and procedures (percutaneous coronary intervention p = 0.04 and coronary angiography p = 0.02). No significant difference was noted in mortality (4.7% vs. 3.7%). The percentage of patients presenting from rural areas declined significantly during the COVID period (18% vs. 14%, p = 0.01). In the subgroup analysis of sex, we noticed a falling trend of intervention performed in females during the COVID period (8.2% male vs. 3.3 % female).

**Conclusions:**

This study shows a significant decline in patients presenting with Type I myocardial infarction (MI) and a decrease in cardiovascular imaging and procedures during the COVID period. There was a significant increase noted in Type II MI.

## Introduction

The COVID-19 pandemic caused by the SARS-CoV-2 virus has brought new changes to the global healthcare system [1]. As of the 17^th^ of May, 2021, 882,928 people have been affected in Pakistan, with an estimated death toll of 19,752 [2]. Worldwide, healthcare delivery is experiencing drastic changes due to the pandemic. As hospitals become overwhelmed with COVID-19 positive patients, there are striking changes in patient presentation patterns to the emergency department (ED) for conditions other than COVID-19. Cardiovascular diseases have remained the most common cause of death globally in the last 15 years [3]. Multiple studies have recently reported a decline in admissions with acute myocardial infarction (AMI) and acute decompensated heart failure during the COVID period [4,5,6,7,8,9]. These trends in presentation and admissions can have wide-ranging implications for public health. LMICs such as Pakistan, with their resource-limited healthcare systems and healthcare inequities, are poorly equipped to deal with the challenges of a global pandemic. Our study investigated cardiovascular disease presentation trends and management during the COVID period and compared them to a similar period in the previous year.

## Methods

### Study Design and Data Collection

We conducted a retrospective study at a major tertiary care hospital in Karachi, with a catchment area of 140,914 square kilometers. It included patients from March-July 2019 (pre-COVID period) and March–July 2020 (COVID period). We reviewed the medical records of all adult patients (18 years and above) who received a cardiology consultation on their presentation to the emergency department (ED) for both periods. We enrolled all the patients (age > 18) consulted for cardiology from emergency department, including those who get admitted, LAMA or discharged from ED. All in-patient consultation for cardiology from other services were excluded from the study. We take into consideration only the first consult in case of multiple consultations generated for the same patient.

We compared the differences in baseline demographics, comorbid conditions, patients’ region of origin, presenting complaint, urgency of consultation, final cardiovascular (CV) diagnosis, and admission unit/floor. A comparison was also made for any cardiovascular interventions done. The region of origin defined as either belonging to Karachi (urban) or other than Karachi (rural). Patients were primarily diagnosed on the basis of physical examination, medical history, and investigations such as EKG and cardiac biomarkers. Cardiac diagnosis was categorized as: ST elevation myocardial infarction (STEMI), Non-ST elevation myocardial infarction (NSTEMI), unstable angina, Type II myocardial injury, stable coronary artery disease, heart failure (including both preserved and reduced ejection fraction), tachyarrhythmia, bradyarrhythmia, conduction blocks, non-cardiac symptoms, non-cardiac chest pain, pre op risk stratification, hypertensive urgency, syncope, cardiogenic shock, valvular heart disease, and infective endocarditis.

Cardiac intervention included coronary angiography, percutaneous coronary intervention (PCI), primary PCI, coronary artery bypass graft (CABG), cardiac implantable electronic device implantation, diagnostic cardiovascular imaging, or medical therapy. Cardiovascular death was defined as death due to myocardial infarction, heart failure, arrhythmia, cardio-embolic stroke, or during a cardiovascular procedure.

Patient disposition from ED included: coronary care unit (CCU), cardiac step-down unit (CSDU), admission to non-cardiovascular services, leave against medical advice (LAMA), or discharge from ED. Consult priority at our institution is divided into ‘Rush Immediate,’ which requires immediate evaluation, ‘Urgent’ within one hour, whereas ‘non-urgent’ in four hours. Our maximum response time for emergency cardiology consultation is four hours as per our hospital policy.

Data was collected by reviewing electronic medical records after obtaining permission from the Ethical Review Committee of the Aga Khan University Hospital, Karachi. We analyzed the data using statistical software STATA (version 14.2). Categorical data was presented as frequency or proportion. For descriptive analysis, categorical data was summarized as frequencies or proportions. Measures of central tendency were computed for continuous variables. For inferential analysis, patient data from the two study periods i.e. pre-COVID period and the COVID period, were compared for all their characteristics using the Pearson Chi2 test (for qualitative variables with greater than five cell frequency) and Fisher’s exact (for qualitative variables with less than five cell frequency). Independent t-test was used to compute the difference after assessing normality. Calculations were done separately for each variable between two patient groups with significant p-value less than or equal to 0.05. Logistic regression univariate and multivariable analysis was done, using maximum likelihood estimation to analyze the relationship between dependent and independent variables.

## Results

A total of 2976 cardiology consults were generated from the ED for patients presenting with cardiovascular complaints during the two study intervals. 2041 (68%) consults were called during pre-COVID period, and 935 (32%) consults were called during COVID period. Common reasons for cardiology consult were acute coronary syndrome (28%), type II MI (13%), non-cardiac chest pain (12%), and congestive heart failure (11%). Consult priority was labeled as ‘rush immediate’ for 8%, ‘Urgent’ for 86%, and ‘non-urgent’ for 6% of the consults.

### Baseline Characteristics

The demographic and clinical characteristics of the patients were as shown in Table 1. No significant differences were noted in age and gender. Patients presenting during the COVID-period were more likely to be hypertensive, diabetic and had chronic kidney disease. The percentage of patients presenting from rural areas declined during the COVID period.

**Table 1:** Baseline characteristics of patients presenting in pre-COVID and COVID period.

Demographics n = 2976	Pre-COVID (Mar–July 2019) n = 2041	COVID (Mar–July 2020) n = 935	P value

Age (Mean ± SD)	60.7 ±15	61.7 ±15	0.24
Gender^α^			
male	1201 (58.8%)	581 (62.1%)	0.09
female	840 (41.1%)	354 (37.8%)	
Area^α^			
Rural area	373 (18.2%)	136 (14.5%)	0.01*
Urban area	1668 (81.7%)	799 (85.4%)	
HTN^α^	1223 (59.9%)	725 (77.5%)	<0.001*
DM^α^	920 (45%)	465 (49.7%)	0.01*
CKD^α^	211 (10.3%)	130 (13.9%)	<0.01*
Stroke^α^	96 (4.7%)	48 (5.1%)	0.31
IHD^α^	497 (24.3%)	258 (27.5%)	0.05
Prior PCI^α^	291 (14.2%)	126 (13.4%)	0.56

^α^ Fisher Exact test.

### Gender Difference

Although there was no gender-based differences in rates of primary PCI (2.8 % vs. 3.1%, p = 0.85), females faced a significant drop in diagnostic coronary angiography (7.02 % vs. 3.9%, P = 0.04), and elective PCI (7.5% vs. 3.3%, p < 0.01) during the COVID period. By using univariate and multi regression analysis we have checked the plausible interaction against gender and time periods for intervention, however no significant interaction was found (p = 0.31).

### Consultation Type and urgency

The number of consultations called with priority of ‘rush immediate’ were 152 pre-COVID vs. 53 COVID (p = 0.07) and of ‘urgent’ were 1796 pre-COVID versus 744 COVID, (p < 0.001), whereas ‘non-urgent’ consults numbered to 93 pre-COVID versus 138 COVID (P < 0.001).

### Diagnosis

Overall, acute coronary syndrome (ACS) presentations were reduced significantly (p = 0.047). There was non-significant reduction in ST elevation Myocardial Infarction, but significant reduction in Type I NSTEMI, Unstable angina and Heart failure. There was rise in type II myocardial injury during the COVID period. No significant difference was observed in both groups for tachy-arrhythmia, brady-arrhythmia, stable coronary artery disease, non-cardiac chest pain, non-cardiac symptoms, valvular heart disease, cardiogenic shock, and syncope. Pre-operative risk stratification consults reduced significantly during COVID (Table 2).

**Table 2:** Cardiology diagnosis in pre-COVID and COVID period.

Diagnosis (n = 2976)	PreCOVID n = 2041(68%)	COVID n = 935(32%)	p Value

STEMI (n = 154)	104 (5%)	50 (5.3%)	p = 0.77
NSTEMI (n = 538)	409 (20%)	129 (13.7%)	P < 0.001
Unstable angina (n = 175)	132 (6.4%)	43 (4.5%)	p = 0.04
Stable CAD (n = 42)	26 (1.2%)	16 (1.7%)	p = 0.34
Type II MI (n = 393)	153 (7.4%)	240 (25.6%)	p < 0.001
Heart Failure (n = 315)	254 (12.4%)	61 (6.5%)	P < 0.001
Tachyarrhythmia (n = 251)	177 (8.6%)	74 (7.9%)	P = 0.49
Brady arrhythmia (n = 99)	66 (3.2%)	33 (3.5%)	P = 0.67
Non-cardiac symptoms (n = 263)	191 (9.3%)	72 (7.7%)	P = 0.13
Non cardiac chest pain (n = 418)	289 (14.1%)	129 (13.7%)	P = 0.79
Pre op risk stratification (n = 129)	99 (4.8%)	30 (3.2%)	P = 0.04
Hypertensive urgency (n = 83)	62 (3%)	21 (2.2%)	P = 0.22
Syncope (n = 66)	39 (1.9%)	27 (2.8%)	P = 0.09
Cardiogenic shock (n = 4)	3 (0.1%)	1 (0.1%)	P = 0.78
Valvular heart disease	22 (1%)	5 (0.5%)	P = 0.14
Infective endocarditis (n = 7)	6 (0.3%)	1 (0.1%)	P = 0.32

### Admission

Out of 2976 consults generated in the ED, 942 (32%) patients in total were admitted to cardiology service over both periods. As compared to admission in other inpatient services (pre-COVID 36.9% vs COVID 42.5%, p < 0.01) admission rates in cardiology service went down significantly in COVID period. This effect was observed in number of admissions to both coronary care (pre-COVID 24.6% vs COVID 17.9%, p < 0.001) and cardiac step-down units (pre-COVID 9.8% vs COVID 7.4%, p = 0.03).

### Procedure

Total cardiology procedures dropped from 39% pre-COVID to 22% during COVID. The drop in diagnostic coronary angiography, elective PCI and cardiac imaging was statistically significant (Table 3).

**Table 3:** Cardiovascular interventions in pre-COVID and COVID period.

Management n = 2976	Pre-COVID n = 2041	COVID n = 935	p value

PCI	174 (8.5%)	60 (6.4%)	p = 0.04
Coronary angiography	129 (6.32%)	29 (3.10%)	P < 0.01
Primary PCI (STEMI n =	82 (4%)	34 (3.6%)	p = 0.61
CABG	75 (3.7%)	32 (3.4%)	p = 0.73
Cardiac Imaging	481 (23.5%)	101 (10.8%)	p < 0.001
Device Implantation	24 (1.2%)	7 (0.7%)	p = 0.48

### Mortality

Overall cardiovascular death was 4.7% with a mean age of 70 ± 7 in the pre-COVID period and 3.7% with a mean age of 66 ±12 during the COVID period.

## Discussion

There are many challenges to the delivery of effective cardiovascular care in LMICs including Pakistan. These include lack of specialized care in rural areas, misdiagnoses, gaps in treatment, gender and ethnic disparities and cost restraints. There is a growing body of data noting the impact of Covid-19 on cardiac services, patient access to care and cardiovascular outcomes from developed countries. However, its effect on a resource-limited, cost-restrained health care system in an LMIC has not been comprehensively reported so far. Our study shows that cardiology services at our tertiary care center were seriously affected by the pandemic.

A statistically significant increase in patients presenting with hypertension, diabetes, and CKD during the COVID period was observed (Table 1). We know from recent data that COVID patients with these comorbidities tend to be sicker with more cardiovascular involvement and, thus, more likely to require ED evaluation and cardiology consultation [10].

Gender differences in both elective and urgent cardiovascular care in the pre-COVID era are well-documented [11,12]. It has been reported that COVID disproportionately affects males, resulting in a more severe illness and higher mortality rates [13,14]. This was supported by the findings in a study from Spain where there was a significant drop in males presenting with STEMI, without a significant change in female numbers [15]. This contrasts with what previously is known from a systemic review of literature from 1960 to 2008 whereby females experienced longer prehospital delay in seeking health care advice on developing symptoms of acute coronary syndrome [16]. In our institution, fewer female patients presented with cardiac complaints during the COVID period, although this did not reach level of statistical significance (Table 2). However, female patients were significantly less likely to undergo elective diagnostic coronary angiography and angioplasty during the pandemic (Table 2). Women in our part of the world face many challenges in accessing timely healthcare due to cultural barriers, reliance on males in the family for transport, lack of education, and prioritizing the health of male family members over their own, just to name a few [17,18,19]. Understandably, all these factors are amplified during the pandemic and lockdowns. No statistically significant difference in inpatient cardiovascular mortality was noted when comparing the pre-COVID and COVID groups.

Our tertiary care university hospital is in Karachi and serves an immediate urban population of 18 million people and a combined urban/rural catchment area of over 140,914 square km [20]. The patients from rural areas using various means of transport (e.g., public, buses, and private cars) sometimes take up to 24 h to reach us. It has been shown that logistics play a major role in access to healthcare in LMICs [21,22]. Due to the pandemic and lockdown, stay-at-home orders, and lack of public transport, we noted a statistically significant drop in patients coming to us from rural areas. Since the incidence of cardiovascular events is unlikely to change, patients from rural areas were delaying coming to a tertiary care center due to fear of the pandemic. Data from Italy’s Lombardi region, which was one of the worst affected areas, showed up to a 150% increase in CV mortality and death due to delayed access to care [23]. Both short and long-term consequences of this delay in cardiovascular care in our rural population remain unmeasured.

As a multispecialty tertiary care hospital, our PCI volume has traditionally consisted of a greater proportion of NSTEMI patients compared to STEMI patients. This has remained true during the COVID period in which most of the patients with acute MI had NSTEMI. An overall decline in both STEMI and NSTEMI numbers identical to other studies was found [5,6,7,8,9], but only reduction in the latter was statistically significant (Table 3). Due to the drop in trend of ACS patients, a statistically significant reduction was seen in ‘Rush immediate’ and ‘Urgent’ consults. Interestingly, of the total patients with raise troponins (above 99^th^ percentile), a sharp rise in Type II MI was seen. Studies have shown rise in cardiac biomarkers in COVID infection which is the most likely reason for the difference seen in our population [23,24,25].

Along with ACS, the number of heart failure patients was also reduced during the COVID period. Reluctance to visit the hospital out of fear of contracting COVID 19 has a major role to play in reduced HF presentation [26]. The other possible cause could be social distancing, which has led to a reduction in respiratory infections, a common trigger for decompensated HF [26]. Hence, fewer HF patients were admitted to the cardiology service during the pandemic, consistent with previously published data [26].

We found an insignificant decrease in both tachy- and bradyarrhythmias during COVID. A recently published study similarly showed a significant reduction in admission with atrial arrhythmia, ventricular arrhythmia and bradyarrhythmia [8]. However, further studies are required in this regard to establish an impact of pandemic on arrhythmia presentation. The significant reduction in preop risk stratification consults for non-cardiac surgeries (pre-COVID 5% versus COVID 3%, p = 0.04) reflects the postponement of elective surgeries as resources were reallocated to meet the needs of the pandemic.

Due to the drop in ACS, we noted a considerable decrease in invasive procedures, especially coronary angiography, and PCI (p = 0.02 and 0.04, respectively), which is a finding similar to data from other studies [6,7]. Contrary to previous studies, no difference was observed in CABG and primary PCI in either period [27]. As with most institutions, we encountered an initial difficulty in setting standard operating procedures for cardiovascular imaging, especially echocardiography. Our cardiovascular imaging volumes, including echo, nuclear stress testing, ETT, cardiac MRI and Holter monitoring suffered large a large decline [5,28]. We expect that as the COVID numbers reach a plateau, patients who delayed their cardiac tests will return, and we will observe a gradual catch-up to pre-COVID volumes. This study has potential limitations. It is a single centered retrospective study and hence lacks generalizability. Data was obtained from electronic medical system which carries its own margin of error. Another limitation to generalization is that the study was carried out in a private sector hospital of the epicenter and hence might not have included population below the poverty line.

## Conclusion

This study from a low to middle income country highlights the significant drop in cardiovascular disease presentation and admissions during the pandemic. The results are consistent to western literature indicating similar epidemiological impact. However, there are indications that the impact has been disproportionate for women and patients from rural regions. These were already at-risk populations pre-pandemic and focused efforts need to be made to improve access to cardiovascular care for them. Health care authorities need to be cognizant of the fallout from delayed cardiac care and prepare for future burden of increased cardiovascular morbidity and mortality.

## Data Accessibilty Statement

Data are available on reasonable request.

## References

[1] Mareiniss DP. The impending storm: COVID-19, pandemics and our overwhelmed emergency departments. The American Journal of Emergency Medicine. 23 March 2020. DOI: 10.1016/j.ajem.2020.03.033 PMC7102611 32253132

[2] Government of Pakistan. Government of Pakistan Health Advisory Platform; 2020.

[3] World Health Organization (WHO). The top 10 causes of death; 2020.

[4] Bhatt AS, Moscone A, McElrath EE, et al. Declines in hospitalizations for acute cardiovascular conditions during the COVID-19 pandemic: A multicenter tertiary care experience. Journal of the American College of Cardiology. 2020; 27379. DOI: 10.1016/j.jacc.2020.05.038 PMC7250561 32470516

[5] Fersia O, Bryant S, Nicholson R, et al. The impact of the COVID-19 pandemic on cardiology services. Open Heart. 2020; 7: e001359. DOI: 10.1136/openhrt-2020-001359 32855212 PMC7454176

[6] Mafham MM, Spata E, Goldacre R, et al. COVID-19 pandemic and admission rates for and management of acute coronary syndromes in England. Lancet. 8 August 2020; 396(10248): 381–389. DOI: 10.1016/S0140-6736(20)31356-8. Epub 2020 Jul 14. PMID: 32679111; PMCID: PMC7429983. 32679111 PMC7429983

[7] Enache B, Claessens YE, Boulay F, et al. Reduction in cardiovascular emergency admissions in Monaco during the COVID-19 pandemic. Clin Res Cardiol. 2020; 109(12): 1577–1578. DOI: 10.1007/s00392-020-01687-w 32533248 PMC7291940

[8] Sokolski M, Gajewski P, Zymliński R, et al. Impact of Coronavirus Disease 2019 (COVID-19) Outbreak on Acute Admissions at the Emergency and Cardiology Departments Across Europe. Am J Med. 30 September 2020; S0002-9343(20)30825-1. DOI: 10.1016/j.amjmed.2020.08.043. Epub ahead of print. PMID: 33010226; PMCID: PMC7526639. PMC7526639 33010226

[9] Vacanti G, Bramlage P, Schymik G, et al. Reduced rate of admissions for acute coronary syndromes during the COVID-19 pandemic: An observational analysis from a tertiary hospital in Germany. Herz. 2020; 45: 663–667. DOI: 10.1007/s00059-020-04991-3 33026483 PMC7539285

[10] Rashid Hons M, Gale Hons CP, Curzen Hons N, et al. Impact of Coronavirus Disease 2019 Pandemic on the Incidence and Management of Out-of-Hospital Cardiac Arrest in Patients Presenting With Acute Myocardial Infarction in England. J Am Heart Assoc. 17 November 2020; 9(22): e018379. DOI: 10.1161/JAHA.120.018379. Epub 7 October 2020. PMID: 33023348; PMCID: PMC7763705. 33023348 PMC7763705

[11] Nante N, Messina G, Cecchini M, et al. Sex differences in use of interventional cardiology persist after risk adjustment. Journal of Epidemiology & Community Health. 2009; 63: 203–208. DOI: 10.1136/jech.2008.077537 19052034 PMC2635953

[12] Sciomer S, Moscucci F, Dessalvi CC, Deidda M, Mercuro G. Gender differences in cardiology: Is it time for new guidelines? Journal of Cardiovascular Medicine. December 2018; 19(12): 685–688. DOI: 10.2459/JCM.0000000000000719 30239478

[13] Jin J, Bai P, He W, Wu F, et al. Gender differences in patients with COVID-19: Focus on severity and mortality. Journal; 2020. DOI: 10.1101/2020.02.23.20026864 PMC7201103 32411652

[14] Guan WJ, Ni ZY, Hu Y, et al. Clinical characteristics of coronavirus disease 2019 in China. N Engl J Med. 2020; 382: 1708–20. DOI: 10.1056/NEJMoa2002032 32109013 PMC7092819

[15] Moreno R, et al. ‘Influence of age and gender on arrival of patients with ST-segment elevation acute myocardial infarction to tertiary centers during COVID-19 pandemic. Experience of Madrid, Spain, STEMI network (Codigo Infarto Madrid).’ The American journal of emergency medicine. 6 June 2020; S0735-6757(20)30494-0. DOI: 10.1016/j.ajem.2020.06.013

[16] Nguyen HL, Saczynski JS, Gore JM, Goldberg RJ. Age and sex differences in duration of prehospital delay in patients with acute myocardial infarction: A systematic review. Circ Cardiovasc Qual Outcomes. January 2010; 3(1): 82–92. DOI: 10.1161/CIRCOUTCOMES.109.884361. Epub 24 November 2009. PMID: 20123674; PMCID: PMC3072277. 20123674 PMC3072277

[17] Assiri AS. Gender differences in clinical presentation and management of patients with acute coronary syndrome in Southwest of Saudi Arabia. J Saudi Heart Assoc. July 2011; 23(3): 135–41. DOI: 10.1016/j.jsha.2011.01.007. Epub 9 January 2011. PMID: 24146527; PMCID: PMC3801142. 24146527 PMC3801142

[18] Lu HT, Nordin R, Wan Ahmad WA, et al. Sex differences in acute coronary syndrome in a multiethnic asian population: Results of the malaysian national cardiovascular disease database-acute coronary syndrome (NCVD-ACS) registry. Glob Heart. December 2014; 9(4): 381–90. DOI: 10.1016/j.gheart.2014.06.001. PMID: 25592791. 25592791

[19] Altaf A, Shah H, Salahuddin M. Gender based differences in clinical and Angiographic characteristics and outcomes of Acute Coronary Syndrome (ACS) in Asian population. Pak J Med Sci. September–October 2019; 35(5): 1349–1354. DOI: 10.12669/pjms.35.5.743. PMID: 31489005; PMCID: PMC6717489. 31489005 PMC6717489

[20] http://www.pbs.gov.pk/content/provisional-summary-results-6th-population-and-housing-census-2017-0.

[21] Varela C, Young S, Mkandawire N, et al. Transportation barriers to access health care for surgical conditions in Malawi: A cross-sectional nationwide household survey. BMC Public Health. 2019; 19; 264. DOI: 10.1186/s12889-019-6577-8 30836995 PMC6402149

[22] Akhlaq A, McKinstry B, Muhammad KB, Sheikh A. Barriers and facilitators to health information exchange in low- and middle-income country settings: A systematic review. Health Policy and Planning. November 2016; 31(9): 1310–1325. DOI: 10.1093/heapol/czw056 27185528

[23] Odone A, et al. ‘COVID-19 deaths in Lombardy, Italy: data in context.’ The Lancet Public health. 2020; 5(6): e310. DOI: 10.1016/S2468-2667(20)30099-2 32339478 PMC7182509

[24] Babapoor-Farrokhran S, Gill D, Walker J, Rasekhi RT, Bozorgnia B, Amanullah A. Myocardial injury and COVID-19: Possible mechanisms. Life sciences. 2020; 253: 117723. DOI: 10.1016/j.lfs.2020.117723 32360126 PMC7194533

[25] Shi S, Qin M, Cai Y, et al. Characteristics and clinical significance of myocardial injury in patients with severe coronavirus disease 2019. European Heart Journal. 2020; 41(22): 2070–9. DOI: 10.1093/eurheartj/ehaa408 32391877 PMC7239100

[26] Bromage DI, Cannata A, Rind IA, et al. The impact of COVID-19 on heart failure hospitalization and management: Report from a Heart Failure Unit in London during the peak of the pandemic. European Journal of Heart Failure; June 2020. DOI: 10.1002/ejhf.1925 PMC7300902 32478951

[27] Fersia O, Bryant S, Nicholson R, et al. The impact of the COVID-19 pandemic on cardiology services. Open Heart. August 2020; 7(2): e001359. DOI: 10.1136/openhrt-2020-001359. PMID: 32855212; PMCID: PMC7454176 32855212 PMC7454176

[28] Zoghbi WA, DiCarli MF, Blankstein R, et al. Multimodality Cardiovascular Imaging in the Midst of the COVID-19 Pandemic: Ramping Up Safely to a New Normal. JACC Cardiovasc Imaging. 2020; 13(7): 1615–1626. DOI: 10.1016/j.jcmg.2020.06.001 32646721 PMC7290215

